# Playing sport injured is associated with osteoarthritis, joint pain and worse health-related quality of life: a cross-sectional study

**DOI:** 10.1186/s12891-020-3136-5

**Published:** 2020-02-19

**Authors:** Garrett S. Bullock, Gary S. Collins, Nick Peirce, Nigel K. Arden, Stephanie R. Filbay

**Affiliations:** 1 Centre for Sport, Exercise and Osteoarthritis Research Versus Arthritis, University of Oxford, Oxford, UK; 2 Nuffield Department of Orthopaedics, Rheumatology, and Musculoskeletal Sciences, University of Oxford, B4495, Oxford, OX3 7LD UK; 3 Centre for Statistics in Medicine, Nuffield Department of Orthopaedics, Rheumatology, and Musculoskeletal Sciences, University of Oxford, Oxford, UK; 4 Oxford University Hospitals NHS Foundation Trust, Oxford, UK; 5 England and Wales Cricket Board, National Cricket Performance Centre, Loughborough University, Loughborough, LE11 3TU UK; 6 National Centre for Sport and Exercise Medicine, Loughborough University, Loughborough, LE11 3TU UK

**Keywords:** Recreational sport, Former athletes, Mental health, Musculoskeletal health

## Abstract

**Background:**

Sports participants are faced with the decision to continue or cease play when injured. The implications of playing sport while injured on joint health and health-related quality of life (HRQoL) has not been investigated. The purpose of this study was to investigate the relationship between having played sport while injured and HRQoL, osteoarthritis, and persistent joint pain; and compare findings in elite and recreational cricketers.

**Methods:**

The Cricket Health and Wellbeing Study cohort was used for this study. Inclusion criteria were: age ≥ 18 years, played ≥1 cricket season. Questionnaire data collected included a history of playing sport injured, SF-8 (physical (PCS) and mental (MCS) component scores), physician-diagnosed osteoarthritis, and persistent joint pain (most days of the last month). Multivariable linear regressions and logistic regressions were performed. Continuous covariates were handled using fractional polynomials. Models were adjusted for age, sex, cricket-seasons played, playing status, joint injury, and orthopaedic surgery. All participants (*n* = 2233) were included in HRQoL analyses, only participants aged ≥30 years (*n* = 2071) were included in osteoarthritis/pain analyses.

**Results:**

Of the 2233 current and former cricketers (mean age: 51.7 SD 14.7, played 30 IQR 24 cricket seasons, 60% were current cricketers, 62% played recreationally; median PCS: 51.4 IQR 9.0; MCS: 54.3 IQR 8.6) 1719 (77%) had played sport while injured. People who had played sport injured reported worse adjusted PCS (Effect(95% CI): − 1.78(− 2.62, − 0.93) and MCS (− 1.40(− 2.25, − 0.54), had greater odds of osteoarthritis (adjusted OR(95% CI): 1.86(1.39, 2.51) and persistent joint pain (2.34(1.85, 2.96)), compared to people who had not played sport injured. Similar relationships were observed regarding PCS, osteoarthritis and pain in elite and recreational subgroups. Playing injured was only related to worse MCS scores for elite cricketers (− 2.07(− 3.52, − 0.63)); no relationship was observed in recreational cricketers (− 0.70(− 1.79, 0.39)).

**Conclusion:**

Cricketers that had played sport injured had impaired HRQoL, increased odds of osteoarthritis and persistent joint pain, compared to those who had not played sport injured. Playing sport injured was only related to impaired mental-components of HRQoL in elite cricketers. The long-term impact of playing while injured on musculoskeletal health, should be considered when advising athletes on their ability to compete following injury.

## Background

Injuries are a significant concern for athletes [1] and are associated with a substantial economic and physical burden [2]. Sport injury is an established risk factor for developing post-traumatic osteoarthritis (OA), which is associated with higher levels of disability, compared to idiopathic OA [3]. Disability resulting from OA can have detrimental effects on health-related quality of life (HRQoL), [4] and is associated with reduced physical function and persistent pain [5]. In order to inform more effective OA prevention strategies for athletes, an improved understanding of factors, aside from injury, that are related to OA and joint pain in later life is needed. It is evident that some athletes will continue to play sport despite substantial injury, irrespective of the risk of exacerbating the injury, while others will elect to discontinue play following injury. The potential impact of playing sport injured on long-term joint health has not been investigated.

Continuing to compete following an injury can potentially exacerbate injury, and delay an athlete’s functional and performance recovery, [6] compared to ceasing play immediately [7]. Reasons for playing injured include team pressure, [8] fear of losing playing time, [9] significance of upcoming games, [9] and an athlete’s psychological profile [10]. Psychological factors that may relate to playing sport injured include competitiveness, physical and mental coping strategies, and resilience [10]. An athlete’s psychological profile is also associated with wellbeing and HRQoL [11, 12]. For example, wheel-chair basketball athletes with greater resilience reported better HRQoL compared to peers with less resilience [12]. In another study, male and female athletes with greater psychological hardiness and resilience reported improved psychological wellbeing compared to athletes with lower psychological hardiness and resilience [11]. Despite the negative implications for joint health; individuals who play sport while injured may possess psychological characteristics that reduce the impact of OA and pain on HRQoL in later life. However, the relationship between playing sport while injured and HRQoL is poorly understood.

Elite and recreational athletes potentially play while injured at different rates [13]. Specifically, lower level football players competed more often and for longer while injured compared to professional football players [13]. The differences seen in the amount of time these athletes play while injured have been attributed to resource availability and motivations [13]. Athletes competing at an elite standard have greater individual and organizational resources available, allowing for teams to better handle time loss from injury compared to lower standards-of-play [13]. Elite athletes also have different playing motivations, focusing on loss of performance, rather than the overall ability to play, as the determining factor to play while injured [13]. Additionally, recreational sport participants may have more autonomy over deciding whether to continue playing while injured compared to elite athletes, where such decisions may be made by the coaching and medical staff. The contrasting influences on playing sport while injured amongst elite and recreational athletes warrant further consideration.

Currently, it is not understood whether playing sport while injured effects an athlete’s long-term HRQoL or the prevalence of joint pain, and OA. Therefore, the purpose of this study was to i) investigate the relationship between playing sport while injured and HRQoL (physical and mental components); ii) determine the odds of physician-diagnosed OA and persistent joint pain in people who had played sport while injured; iii) compare findings between elite and recreational cricket participants.

## Methods

### Study design

This study was a cross-sectional research design. The Cricket Health and Wellbeing Study was approved by the NHS Health Research Authority (NRES), London Stanmore Research Ethics Committee (REC 15/LO/1274).

### Participants and recruitment

**Fig. 1:**
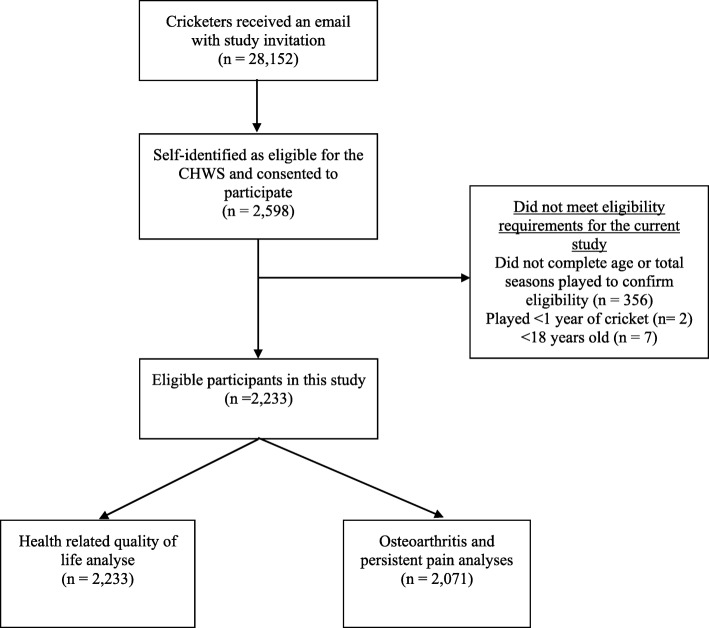
Study flow chart

On March 2017, 28,152 current and former cricketers from all standards-of-play who were registered on the England and Wales Cricket Board national database, were invited by email to complete an electronic questionnaire. Two thousand five hundred ninety-eight cricketers self-identified as meeting the eligibility criteria and gave written consented to participate in the Cricket Health and Wellbeing Study. Participants were eligible for inclusion in the Cricket Health and Wellbeing Study if they had played ≥1 cricket season and were aged ≥18 years. Despite consenting to participate, 365 did not meet eligibility criteria. A total of 2233 cricketers were included in the HRQoL analyses. Due to the rarity of OA in individuals less than 30 years of age, [5] only participants aged ≥30 years (*n* = 2071) were included in OA and persistent joint pain analyses (Fig. 1).

### Questionnaire design

The Cricket Health and Wellbeing Study questionnaire was designed in collaboration with the England and Wales Cricket Board and piloted on current and former cricketers. Following piloting, small changes to the wording of cricket related questions were applied, to enhance clarity. The questionnaire was designed to evaluate five aspects of health and wellbeing (i. cricket-related injury leading to more than 4 weeks of reduced participation in exercise, training or sport; ii. joint pain and OA; iii. General health and disease prevalence; iv. physical activity; v. resilience, quality of life and flourishing). All participant data was de-identified and encrypted into a RedCap® (Research Electronic Data Capture) software-based database. The RedCap® software [14] used branching logic and allowed participants to save their current progress and complete at a later time. For a full description of the survey questions used within this study, please refer to Appendix 1.

### Outcomes

#### Health-related quality of life

The Short Form 8 (SF-8) was used to assess HRQoL [15]. The SF-8 is a short version of the RAND 36-Item Health Survey (SF-36) 1.0 [16]. The SF-8 is scaled and measured on the same point scale (0–100) as the SF-36, with 0 representing maximum disability and 100 representing no disability [17]. The SF-8 is an 8-item, self-reported HRQoL questionnaire comprising 4 domains that contribute to the Physical Component Score (PCS) (general health, physical functioning, role limitations due to physical health problems, bodily pain) and 4 domains that contribute to the mental component score (MCS) (vitality (energy/fatigue), social functioning, mental health, and role limitations due to emotional problems) [17]. The PCS and MCS scores have high reliability (0.88 and 0.82 respectively) for use in the general United States population [18]. The PCS and MCS scores are calculated using a norm-based scoring algorithm that employs a linear T-score transformation with a mean of 50 and a standard deviation of 10, derived from 1998 United States general population norms. For summary measures, group mean scores below 47 can be interpreted as being below the average range for the general population [18]. The minimum detectable difference (MDC) for the PCS was found to be two points in a sample with lower extremity OA, [19] and the minimum clinically important difference (MCID) in the general population has been estimated to range from three to five points for the PCS and MCS [20].

#### Physician-diagnosed osteoarthritis

Osteoarthritis was assessed with the following question, ‘*Have you ever been told by a doctor that you have osteoarthritis (wear and tear or joint degeneration)?’*

#### Persistent joint pain

Persistent joint pain was assessed with the following question, *‘Have you had pain in your [left/right] [hip/groin, knee, ankle, shoulder, hand/finger, spine/back, other joint] on most days of the last month?’*

### Explanatory variables

#### History of playing sport while injured

Participants responded to the following question, ‘*Have you ever played sport injured, despite feeling like doing so might make the injury worse?*’ Response options included ‘yes’, ‘no’, or ‘don’t know’. ‘Don’t know’ responses were excluded from the analyses. There was a total of 54 ‘don’t know’ responses, with no differences in participant characteristics between participants that responded ‘yes’, ‘no’, or ‘don’t know’.

#### Standard-of-play

Standard of play was assessed with the following question, ‘*What was the highest standard of cricket that you played for at least one season*?’ Response options included: international; county/premier league; academy or county age group; university; school; village or social; don’t know. Participants were stratified into recreational (university, school, village or social) and elite (international or county/premier league, academy or county age group).

### Covariates

Covariates were identified through clinical reasoning and a review of the literature [21–25]. Covariates included age, gender, cricket seasons played, playing status, number of joints injured, and number of orthopaedic joint surgeries. Playing status was assigned as either currently playing cricket (0) or no longer playing cricket [1]. Number of joints injured was assessed with the following question, ‘*Have you ever had any cricket-related injuries leading to more than 4 weeks of reduced participation in exercise, training or sport? If yes, where? Please write the number of injuries for each joint and side*’ Participants were stratified into never sustained a joint injury (0), and sustained a joint injury [1]. Number of orthopaedic surgeries were assessed by asking the following question, ‘*Have you ever had orthopaedic surgery (including bone, ligament or joint surgery)? If yes, where? Please write the number of surgeries for each joint and side.*’ Participants were stratified into never had an orthopaedic surgery (0), and had an orthopaedic surgery [1].

### Statistical analyses

Continuous covariates were not assumed to linearly affect the outcome, and were modelled using fractional polynomials. As a result, multivariable linear regressions with fractional polynomial regressions were used to investigate the relationship between playing sport while injured and HRQoL (MCS and PCS scores) in all participants aged 18 years and over. Unadjusted and adjusted coefficients and 95% confidence intervals (95% CI) were calculated. All assumptions for fractional polynomial regression were evaluated and satisfied [26]. Logistic regression was used to investigate the relationship between playing sport while injured and joint health (physician-diagnosed OA and the presence of persistent joint pain). Unadjusted and adjusted odds ratios (ORs) and 95% CI were calculated. All assumptions for logistic regressions were evaluated and met [27]. All regression models were adjusted for age, cricket seasons played, playing status, number of joints injured, and number of joint surgeries. All analyses were repeated in elite and recreational cricketer subgroups to address the second aim of this study.

Data were assessed for missingness prior to analysis. Missing data were calculated as total number and percentage of total data. Due to the low percentage of missing data (MCS: 6.5% PCS; 6.5%, OA: 1%, persistent joint pain: 1.1%, history of playing while injured: 2.4%, joint injury history: 3.8%, orthopaedic surgery history: 1.0%), complete case analyses were performed. ‘Don’t know’ responses (history of playing sport while injured: *n* = 54 (2.1%), OA: *n* = 67 (2.6%), persistent joint pain: *n* = 12 (0.5%), age: 0 (0%), playing status: 0 (0%), joint injury history: 15 (0.6%), orthopaedic surgery history: 4 (1.6%)) were not included in the regression analyses. All analyses were performed in R version 3.5.1 (R Core Team (2013). R: A language and environment for statistical computing. R Foundation for Statistical Computing, Vienna, Austria. URL http://www.R-project.org/), using the naniar package for missingness assessment, [28] and the mfp package for fractional polynomial regression [29].

## Results

A total of 2233 cricketers (aged mean 51.7 SD 14.7 years, played an average 30 IQR 24 seasons, 60% were current cricketers, 62% had only played recreationally) were included in analyses (Table 1). 1719 (77%) had played sport while injured. The median PCS score was 51.4 (IQR 9.0) and MCS score was 54.3 (IQR 8.6). Cricketers that had played while injured reported a median PCS score of 51.1 (IQR 9.0) and MCS score of 53.8 (IQR 9.3). Cricketers that had not played while injured reported a median PCS score of 52.7 (IQR 8.5) and MCS score of 55.6 (IQR 7.5). Two thousand seventy-one participants were aged ≥30 years and were eligible for inclusion in the OA and persistent joint pain analyses (Table 1). 1324 (65%) of ≥30 year old cricketers reported persistent joint pain and 602 (30%) reported being diagnosed with OA.

**Table 1:** Participant characteristics

	All participants (*n* = 2233)	Never played sport injured (*n* = 508)	Played sport injured (*n* = 1725)	Participants aged ≥30 years (*n* = 2071)	Never played sport injured (*n* = 461)	Played sport injured (*n* = 1556)
Age (years)	51.7 (SD 14.7)	54.5 (SD 15.7)	50.7 (SD 14.2)	54.5 (SD 12.2)	57.6 (SD 13.0)	53.6 (SD 13.0)
Sex						
Male	2215 (97%)	477 (95%)	1680 (97%)	2008 (97%)	436 (95%)	1521 (98%)
Female	65 (3%)	26 (5%)	36 (3%)	51 (3%)	21 (5%)	27 (2%)
BMI (kg/m2)	27.8 (SD 5.0)	27.8 (SD 5.6)	27.9 (SD 4.8)	28.1 (SD 4.9)	28.1 (SD 5.7)	28.0 (SD 4.7)
Cricket Seasons Played	30 (IQR 24)	27 (IQR 28)	35 (IQR 18)	30 (IQR 20)	30 (IQR 28)	31 (IQR 19)
Joints Injured						
0	1207 (53%)	369 (74%)	799 (47%)	1078 (53%)	331 (73%)	714 (47%)
1+	1046 (47%)	130 (26%)	897 (53%)	958 (47%)	122 (27%)	817 (53%)
Orthopaedic Surgeries						
0	1472 (64%)	374 (74%)	1055 (61%)	1291 (62%)	332 (72%)	921 (59%)
1+	797 (35%)	128 (26%)	652 (38%)	761 (37%)	124 (27%)	621 (39%)
Persistent joint pain						
No	848 (37%)	273 (55%)	552 (33%)	728 (35%)	239 (53%)	461 (31%)
Yes	1412 (63%)	229 (45%)	1146 (67%)	1324 (65%)	217 (47%)	1071 (69%)
Physician diagnosed osteoarthritis						
No	1598 (72%)	394 (80%)	1161 (70%)	1391 (70%)	349 (78%)	1005 (67%)
Yes	611 (27%)	98 (20%)	500 (30%)	602 (30%)	98 (22%)	491 (33%)
Standard-of-play						
Elite	872 (39%)	159 (32%)	690 (41%)	754 (37%)	138 (31%)	597 (39%)
Recreational	1363 (61%)	336 (68%)	993 (59%)	1261 (63%)	311 (69%)	918 (61%)
History of 4+ week time loss injury						
No	1195 (53%)	366 (73%)	790 (47%)	1067 (52%)	328 (72%)	706 (46%)
Yes	1058 (47%)	133 (27%)	906 (53%)	969 (48%)	125 (28%)	825 (54%)

^a^ Discrepancies in variable count are due to responses of ‘don’t know’

### The relationship between playing sport while injured and health-related quality of life

#### All participants

After adjusting for all covariates including history of joint injury, participants that had played sport injured reported (co-efficient (95% CI)) -1.78 (− 2.62 to − 0.93) points worse PCS scores, and − 1.40 (− 2.25 to − 0.54) points worse MCS scores than participants who had not played sport while injured (Table 2).

**Table 2:** Linear regression analysis investigating the relationship between playing sport while injured and health related quality of life

	Physical Component Score		Mental Component Score	
	Unadjusted	Adjusted^a^	Unadjusted	Adjusted^a^
	Effect	Effect	Effect	Effect
	(95% CI)	(95% CI)	(95% CI)	(95% CI)
Played sport while injured (*n* = 1725, 77%)	−1.53 (−2.37, −0.69), *P* < 0.001	− 1.78 (− 2.62, − 0.93), *P* < 0.001	−1.49 (− 2.32, − 0.66), *P* < 0.001	− 1.40 (− 2.25, − 0.54), *P* < 0.001
Age^b^		−13.03 (− 17.16, −8.89), *P* < 0.001		0.79 (0.42, 1.17), *P* < 0.001
Age^c^				−5.10 (−7.55, − 2.64), *P* < 0.001
Gender		−1.68 (−3.77, 0.41), *P* = 0.115		−0.09 (− 2.25, 2.21), *P* = 0.934
Cricket Seasons Played^d^		0.46 (0.16, 0.76), *P* = 0.003		0.81 (0.48, 1.13), *P* < 0.001
Playing Status		−2.52 (−3.31, −1.72), P < 0.001		−0.29 (−1.09, 0.55), *P* = 0.490
Joints Injured		−1.60 (−2.30, − 1.00), P < 0.001		−0.77 (− 1.49, − 0.06), *P* = 0.035
Orthopaedic Surgeries		− 2.18 (− 3.00, − 1.46), P < 0.001		0.09 (− 0.09, 1.10), *P* = 0.808

^a^ Estimates were adjusted for age, gender (male = 0, female = 1), cricket seasons played, playing status (current = 0, former = 1), history of joint injury (no joints injured = 0, sustained a joint injury = 1), and history of orthopaedic surgery (never had an orthopaedic surgery = 0, underwent orthopaedic surgery = 1)

^b^ Age was defined as (Age/100)^3 for PCS analyses and (Age/100)^1 for MCS analyses

^c^ Second order fractional polynomial was not used for PCS analyses and Age was defined as (Age/100)^2 for MCS analyses

^d^ Cricket seasons for PCS and MCS analyses were divided by ten (Seasons/10)

^e^ SF-8: Short-Form 8 Health Survey; Physical Component Scores (PCS) were calculated using norm based scoring (population norm 50 SD 10, high scorer = better health-related quality of life); Mental Component Scores (MCS) were calculated using norm based scoring (population norm 50 SD 10, high scorer = better health-related quality of life)

#### Elite vs. recreational cricketers

For both elite and recreational cricketers, having played sport while injured was associated with worse PCS scores ((co-efficient (95% CI)) Elite: − 1.64 (− 3.09 to − 0.20); Recreational: -1.89 (95% CI: − 2.94 to − 0.83)), after adjustment for covariates (Table 3). Elite cricketers that had played sport while injured reported − 2.07 (− 3.52 to − 0.63) points worse MCS scores compared to elite cricketers that had not played sport while injured. Having played sport while injured was not related to MCS scores in recreational cricketers (− 0.70 (− 1.79 to 0.39)) (Table 3).

**Table 3:** Linear regression analysis investigating the relationship between playing sport while injured and health related quality of life, in elite and recreational cricketer subgroups

	Physical Component Score		Mental Component Score	
	Unadjusted Effect (95% CI)	Adjusted^a^ Effect (95% CI)	Unadjusted Effect (95% CI)	Adjusted^a^ Effect (95% CI)
Elite (*n* = 849)				
Played sport while injured (*n* = 690, 81%)	− 2.06 (− 3.52–0.59), *P* = 0.006	−1.64 (− 3.09, − 0.20), *P* = 0.026	− 2.27 (− 3.68, − 0.87), *P* = 0.002	− 2.07 (− 3.52, − 0.63), *P* = 0.005
Never played sport while injured (*n* = 159, 19%)	*Reference Group*			
Recreational (*n* = 1329)				
Played sport while injured (*n* = 993, 75%)	− 1.42 (− 2.46, − 0.38), *P* = 0.008	− 1.89 (− 2.94, − 0.83), *P* < 0.001	−0.93 (− 1.98, 0.12), *P* = 0.084	−0.70 (− 1.79, 0.39), *P* = 0.208
Never played sport while injured (*n* = 336, 25%)	*Reference Group*			

^a^ Estimates were adjusted for age, gender (male = 0, female = 1), cricket seasons played, playing status (current = 0, former = 1), history of joint injury (no joints injured = 0, sustained a joint injury = 1), and history of orthopaedic surgery (never had an orthopaedic surgery = 0, underwent orthopaedic surgery = 1)

^b^ SF-8: Short-Form 8 Health Survey; Physical Component Scores (PCS) were calculated using norm based scoring (population norm 50 SD 10, high scorer = better health-related quality of life); Mental Component Scores (MCS) were calculated using norm based scoring (population norm 50 SD 10, high scorer = better health-related quality of life)

### The odds of reporting physician-diagnosed osteoarthritis and persistent joint pain in people who had played sport while injured

#### All participants

Participants aged ≥30 years who had played sport while injured had 1.86 (95% CI 1.39 to 2.51) times greater odds of reporting being diagnosed with OA compared to those that had not played while injured, after adjustment for covariate factors (Table 4). Cricketers that had played while injured had 2.34 (1.85 to 2.96) times greater odds of reporting persistent joint pain compared to those that had not played while injured, after adjusting for joint injury and other covariates (Table 4).

**Table 4:** Logistic regression analysis investigating the odds of physician diagnosed osteoarthritis and persistent joint pain in people who had played sport while injured

	Physician Diagnosed Osteoarthritis		Presence of Persistent Joint Pain	
	Unadjusted Odds Ratio (95% CI)	Adjusted^a^ Odds Ratio (95% CI)	Unadjusted Odds Ratio (95% CI)	Adjusted^a^ Odds Ratio (95% CI)
Played sport while injured (*n* = 1725, 77%)	1.74 (1.36, 2.24), *P* < 0.001	1.86 (1.39, 2.51), *P* < 0.001	2.56 (2.07, 3.17), *P* < 0.001	2.34 (1.85., 2.96), *P* < 0.001
Age		1.05 (1.04, 1.07), P < 0.001		1.01 (0.99, 1.02), *P* = 0.205
Gender		2.05 (0.96, 4.27), *P* = 0.059		1.91 (0.98, 3.88), *P* = 0.065
Cricket Seasons Played		1.00 (0.99, 1.01), *P* = 0.352		1.00 (0.99, 1.01), P = 0.352
Playing Status		1.27 (0.94, 1.57), *P* = 0.131		1.23 (0.98, 1.54), *P* = 0.078
Joint Injury		1.44 (1.14, 1.81), *P* = 0.001		1.50 (1.23, 1.85), P < 0.001
Orthopaedic Surgery		5.15 (4.12, 6.44), P < 0.001		2.16 (1.75, 2.68), P < 0.001

^a^ Estimates were adjusted for age, gender (male = 0, female = 1), cricket seasons played, playing status (current = 0, former = 1), history of joint injury (no joints injured = 0, sustained a joint injury = 1), and history of orthopaedic surgery (never had an orthopaedic surgery = 0, underwent orthopaedic surgery = 1)

^b^ Physician diagnosed osteoarthritis was defined as having received a previous osteoarthritis diagnosis from a general practitioner

^c^ Persistent joint pain was assessed by asking individuals if they had joint-specific pain on ‘most days of the last month’

#### Elite vs. recreational cricketers

People who had played cricket at either an elite or recreational standard, who had played sport while injured, had greater odds of having received an OA diagnosis compared to those who had not play while injured (Elite: OR (95% CI): 2.12 (1.27 to 3.62); Recreational: 1.58 (1.10 to 2.28)), after adjusting for covariates (Table 5). Both elite and recreational cricketers had greater odds of reporting persistent joint pain if they had played sport while injured (Elite: 2.49 (1.66 to 3.74); Recreational: 2.28 (1.70 to 3.06)) compared to those who had not played sport while injured, after adjustment for covariates.

**Table 5:** Logistic regression analysis investigating the odds of physician diagnosed osteoarthritis and persistent joint pain in people who play sport while injured, in elite and recreational cricketer subgroups

	Physician Diagnosed Osteoarthritis		Presence of Persistent Joint Pain	
	Unadjusted Odds Ratio (95% CI)	Adjusted^a^ Odds Ratio (95% CI)	Unadjusted Odds Ratio (95% CI)	Adjusted^a^ Odds Ratio (95% CI)
Elite (n = 849)				
Played sport while injured (*n* = 690, 81%)	2.42 (1.57, 3.85), *P* < 0.001	2.12 (1.27, 3.62), *P* = 0.004	2.92 (2.00, 4.28), *P* < 0.001	2.49 (1.66., 3.74), *P* < 0.001
Never played sport while injured (n = 159, 19%)	*Reference Group*		*Reference Group*	
Recreational (*n* = 1329)				
Played sport while injured (*n* = 993, 75%)	1.37 (1.02, 1.87), *P* = 0.042	1.58 (1.10, 2.28), *P* = 0.014	2.33(1.79, 3.03), *P* < 0.001	2.28 (1.70., 3.06), *P* < 0.001
Never played sport while injured (n = 336, 25%)	*Reference Group*		*Reference Group*	

^a^ Estimates were adjusted for age, gender (male = 0, female = 1), cricket seasons played, playing status (current = 0, former = 1), history of joint injury (no joints injured = 0, sustained a joint injury = 1), and history of orthopaedic surgery (never had an orthopaedic surgery = 0, underwent orthopaedic surgery = 1)

^b^ Physician diagnosed osteoarthritis was defined as having received a previous osteoarthritis diagnosis from a general practitioner

^c^ Persistent joint pain was assessed by asking individuals if they had joint-specific pain on ‘most days of the last month’

## Discussion

People who had played sport while injured had worse HRQoL (lower PCS and MCS scores), and greater odds of OA and persistent joint pain, compared to people who had never played sport while injured. Having played sport while injured, was related to worse physical-components of HRQoL, a greater odds of OA and persistent joint pain in both recreational and elite cricketer subgroups.

People who had played sport while injured had impaired HRQoL compared to people who never played sport while injured. Although the point estimates and 95% CI’s were below the estimated MCID of three to five points, the MCID was estimated in a general population sample, and may not be representative of a sporting population [20]. Therefore, we cannot dismiss the possibility that observed differences may be clinically relevant. Previous research has observed that sport injuries can impair HRQoL [4].. Similarly, within the current study, multivariate analyses demonstrated that a history of joint injury and orthopaedic surgery were related to worse HRQoL. Further, even after adjusting for these factors, the relationship between playing sport injured and HRQoL remained. Interestingly, despite a high prevalence of persistent joint pain and OA, the mean PCS and MCS scores for individuals who had played sport while injured were 1.8 and 3.3 points above the population normative average, [30] which supports previous former cricketer research [22].

Considering the MCID of three to five points, [20] PCS scores were similar to the general population. In the contrast, cricket players reported greater MCS scores than the general population and these exceeded the estimated MCID. These findings support a previous meta-analysis, in which former athletes with impaired PCS scores reported greater MCS scores compared to the population norm [31]. This suggests that psychological factors may positively impact HRQoL in sport participants, despite an increased odds of OA and persistent joint pain.

One possible explanation for this study finding no meaningful impairment in HRQoL in individuals who had played sport injured, despite increased odds of OA and persistent joint pain, is that current and former cricketers have greater levels of resilience compared to the general population [32, 33]. Resilience is defined as an individual’s ability to positively adapt to stress and challenges [34]. Contributing factors to resilience include mental toughness and coping ability [32, 33]. Resilience was found to be an important determinant of wellbeing in former elite cricketers, who reported high satisfaction with quality of life despite living with pain and OA [25]. Additionally, resilience and effective pain coping strategies enabled former cricketers to maintain a physically active lifestyle despite joint pain and physical impairment, with important implications for wellbeing [35]. Sports participants who play while injured may be better at handling the adversity and stress related to sustaining an injury, and the challenges of living with joint pain and OA in later life [32, 33]. This could partly explain why mental-components of HRQoL were not impaired in former cricketers who had played injured compared to the general population, despite impaired PCS scores, a higher odds of OA and persistent joint pain.

Cricketers who had played sport while injured had greater odds of having physician-diagnosed OA and persistent joint pain. Persistent joint pain is the most debilitating OA symptom, and is the foremost reason for patients to seek medical advice for OA [36]. Although the relationship between joint injury and OA is well established, [3, 37] this relationship remained after adjusting for covariates including a history of joint injury and orthopaedic surgery. Playing sport while injured may expose athletes to repeated trauma and heighten the risk of subsequent injury or injury exacerbation, which may predispose an athlete to joint pain and post-traumatic OA [38]. Chronic repeated mechanical stress has been shown to decrease articular cartilage thickness and increase OA progression [39]. Furthermore, sport participation entails fast high impact movements, [40] and when performed on misaligned or injured joints, can increase joint degradation and OA prevalence [41]. Persistent joint pain and OA have been shown to restrict physical function and limits one’s ability to perform preferred physical activity and sports [5]. Our findings highlight the importance of informing both elite and recreational sports participants on the potential long-term joint health ramifications of playing sport while injured.

Elite and recreational cricketers participants who had played sport while injured both had reduced physical-components of HRQoL, an increased odds of OA, and persistent joint pain. However, only elite athletes that competed while injured had reduced mental-components of HRQoL. One possible explanation is the differences in elite and recreational athletes ability to decide when it is appropriate to play with an injury. Recreational sports participants have less medical and organizational support than elite athletes, [13] allowing for increased internal locus of control when deciding to play [42]. Increased locus of control can have a positive effect on mental health and mental-components of HRQoL [42]. This is in contrast to elite athletes that have less medical decision autonomy, and further organizational pressure, leading to higher external locus of control when deciding to play [11]. Further prospective research is needed to investigate the short and long term mental implications of playing sport while injured, across all standards of sport participation.

These results have possible clinical and educational implications for both recreational and elite athletes. Playing sport while injured has potential long-term musculoskeletal implications, at all standards-of-play. While the extent of the injury, competition stresses, [9] team and organization pressure, [8] and athlete competitive spirit [10] may be integrated into clinical decision making, clinicians should weigh the possibility of poorer long-term joint health and outcomes when advising if an athlete should remain or discontinue playing sport following injury. Further, increasing neuromuscular control and strength can improve function in degenerative joints, [43, 44] while a balanced diet and maintaining a healthy wait can help mitigate further OA symptoms [45]. Educating athletes who play sport while injured about potential strategies to reduce the likelihood of developing OA, and to reduce the severity of OA symptoms if the condition develops, could have important health implications in later life. Long-term ongoing physical activity, strength training, and diet should be considered when advising athletes as they transition away from competitive sport [43, 45].

These findings spark future research. It is assumed that athletes understand the long-term implications of playing sport while injured; however, athlete knowledge on this issue is not currently known. Research is required to investigate athlete knowledge concerning the long-term consequences of playing sport while injured. Further, it is currently not known what are effective interventions to reduce the prevalence of athletes playing sport while injured. Deciphering effective organizational, clinical, and athlete-focused interventions may help reduce the prevalence of this issue, while maintaining competitive advantages. Finally, better understanding the long term consequences of playing sport while injured between elite and recreational athletes will allow clinicians and athletes to make better informed decisions when deciding if an athlete should continue to play following an injury.

This was the first study to investigate the relationship between playing sport while injured and HRQoL, OA and persistent joint pain. Missing data was low, reducing bias in these analyses. This study utilized non-linear analyses when appropriate, allowing for a more precise investigation of the true nature of the included variables. Serious injury requiring at least 1 month of time loss from exercise was adjusted for in the analyses. Recalling more serious injuries reduced recall bias (more severe musculoskeletal injuries are associated with a more accurate injury recall [46]). However, more minor injuries (i.e. resulting in less than 1 month of time loss) and soft tissue injuries could have affected the observed relationships. Further, having participants answer questions regarding the past could institute recall bias in these findings. Specifically, osteoarthritis was assessed through patient recall, and not through other avenues such as imaging. Also, the odds of reporting OA and persistent joint pain due to playing injured were not connected to specific joints, but to all joints, decreasing the precision and interpretability in these findings. Potential participants were informed of the inclusion and exclusion criteria during recruitment, and were able to self-select eligibility to participate. Due to this recruitment strategy, it is not possible to determine the questionnaire response rate, hindering the ability to understand participant selection bias.

## Conclusions

After adjusting for joint injury and other covariates, having played sport injured was associated with increased odds of being diagnosed with OA and reporting persistent joint pain, compared to having not played sport injured. Individuals who had played sport while injured also had worse HRQoL than their counterparts who had not; however, this may not be clinically meaningful. Elite and recreational cricketers that had played sport while injured both had impaired physical-components of HRQoL, and increased odds of OA and persistent joint pain. However, only elite athletes who had played sport while injured had reduced mental-components of HRQoL. The long-term impact of playing while injured on musculoskeletal health should be considered when advising athletes on their ability to compete following injury.

## Supplementary information

**Additional file 1: Appendix 1**. Questions and potential responses

## Data Availability

Data is available upon reasonable request to the authors.

## Abbreviations

95% CI: 95% Confidence Interval
HRQoL: Health-related Quality of Life
IQR: Inter Quartile Range
MCID: Minimum Clinical Important Difference
MCS: Mental Component Scores
MDC: Minimal Detectable Change
OA: Osteoarthritis
OR: Odds Ratios
PCS: Physical Component Scores
SF-36: Short Form-36
SF-8: Short Form-8
