# Supplementary material for: Playing sport injured is associated with osteoarthritis, joint pain and worse health-related quality of life: a cross-sectional study
Source: BMC Musculoskelet Disord. 2020 Feb 19;21:111. doi: 10.1186/s12891-020-3136-5 (PMC7031986; doi:10.1186/s12891-020-3136-5)
Supplement: Supplementary file 1 — Additional file 1: Appendix 1. Questions and potential responses [file 12891_2020_3136_MOESM1_ESM.docx]

Appendix 1. Questions and potential responses

| **Construct** | **Item** | **Response** |
| --- | --- | --- |
| Personal Details | Gender: | Male, Female, Other |
|  | Date of birth | Day, Month, Year |
|  | Weight: | Open text box |
|  | Height: | Open text box |
| Cricket History | Playing status: | Currently playing cricket, No longer playing cricket, Plan to return to cricket |
|  | If *no longer* playing cricket what age were you when you played your *last* cricket match? | ___________ (years old),  Don’t know |
|  | What age were you when you played your *first* cricket match? | ___________ (years old), Don’t know |
|  | What is/was your predominant position(s) of play? (select all that apply): | Bowling, Batting, All-rounder, Wicketkeeper, Don’t know |
|  | Approximately how many seasons have you played cricket for? | Number of seasons: _______________ |
|  | What was the highest standard of cricket that you played for at least one season? (please select only one) | International, Count/Premier league, Academy or county age group, University, School, Village of social, Don’t know |
| Pain, injury and surgery | Have you ever had orthopedic surgery (including bone, ligament or joint surgery)? | Yes, No, Don’t know |
|  | If yes, where? Please write the number of surgeries for each joint and side eg Hip left (L / 3, right (R / 0) | Hip, Knee, Ankle, Shoulder, Hand/finger, Spine/back  Other joints: Please specify: ___________ |
|  | Have you ever had any cricket-related injuries leading to more than *4 weeks* of reduced participation in exercise, training or sport? | Yes, No, Don’t know |
|  | If yes, where? Please write the number of injuries for each joint and side  (eg Hip left (L / 3, right (R / 1) | Hip, Knee, Ankle, Shoulder, Hand/finger, Spine/back  Other joints: Please specify: ___________ |
|  | Do you currently experience pain, discomfort, or have any problems in any of your joints? | Yes, No, Don’t know |
|  | If yes, where? Please select all that apply and indicate which side(s) | Hip, Knee, Ankle, Shoulder, Hand/finger, Spine/back  Other joints: Please specify: ___________  If yes, have you had pain on most days of the last month? |
|  | Have you ever been told by a doctor that you have osteoarthritis (wear and tear or joint degeneration)? | Yes, No, Don’t know  Hip, Knee, Ankle, Shoulder, Hand/finger, Spine/back  Other joints: Please specify: ___________ |
|  | Have you ever played sport injured, despite feeling like doing so might make the injury worse? | Yes, No, Don’t know |
| Health Related Quality of Life | Short Form 8 Questionnaire |  |
